# The Impact of Patient-Centered Care in Bipolar Disorder: An Opinion on Caregivers’ Quality of Life

**DOI:** 10.3390/jcm14072209

**Published:** 2025-03-24

**Authors:** Jelena Milic, Iva Zrnic, Milica Vucurovic, Edita Grego, Dragana Jovic, Veroslava Stankovic, Rosa Sapic

**Affiliations:** 1 Institute of Public Health of Serbia “Dr Milan Jovanović Batut”, 11211 Belgrade, Serbia; milica_vucurovic@batut.org.rs (M.V.); grego_edita@batut.org.rs (E.G.);; 2 European Faculty “Kallos”, 11000 Belgrade, Serbia; 3 Regional Medical Chamber of Belgrade, 11111 Belgrade, Serbia; zrniciva1@gmail.com; 4 The College of Health Science, Academy of Applied Studies, 11000 Belgrade, Serbia; stankovicveroslava@gmail.com; 5 Faculty of Health Studies, University of Bjeljina, 76300 Bjeljina, Republika Srpska, Bosnia and Herzegovina; sapicdr@gmail.com

**Keywords:** bipolar disorder, caregivers, mood episodes, patient-centered care, psychotherapeutic and educational strategies, quality of life

## Abstract

**Background/Objectives**: Our background comprises the previously identified consequences of bipolar disorder’s chronicity, which significantly affects not only the patients but also their caregivers, leading to challenges in caregivers’ physical, emotional, and financial well-being. This impact on caregivers’ quality of life (QOL) is often overlooked in the context of patient-centered care for bipolar disorder. Our objective was to explore how patient-centered care in bipolar disorder management affects caregivers, with a focus on improving both patient and caregiver outcomes. **Methods**: We performed a thematic qualitative synthesis of reviewed literature and case studies to explore the intersection of patient-centered care in bipolar disorder management and its impact on caregivers. This comprehensive review allowed us to identify key behavioral patterns and emotional fluctuations in patients that significantly affect caregiver well-being, with a focus on the challenges caregivers face due to the unpredictability of mood episodes and the lack of adequate support in current care models. **Results**: In the results, we identified that while patient-centered care enhances patient outcomes, it also exacerbates the strain on caregivers if their needs are not adequately addressed. Specific behavioral pathways and emotional fluctuations in bipolar disorder impact caregivers, such as the unpredictability of mood episodes, cognitive distortions during manic and depressive phases, and the emotional toll of managing crises. This article also emphasizes the role of patient-centered care, which places the patient at the core of treatment decisions, but often neglects the strain placed on caregivers. **Conclusions**: We conclude that a holistic approach to care, which includes caregiver support and resources, is essential for improving the QOL of both patients and caregivers. Future research is needed to develop strategies and interventions that better support caregivers and enhance their overall well-being, ensuring that patient care models are truly comprehensive.

## 1. Introduction

**Figure 1:**
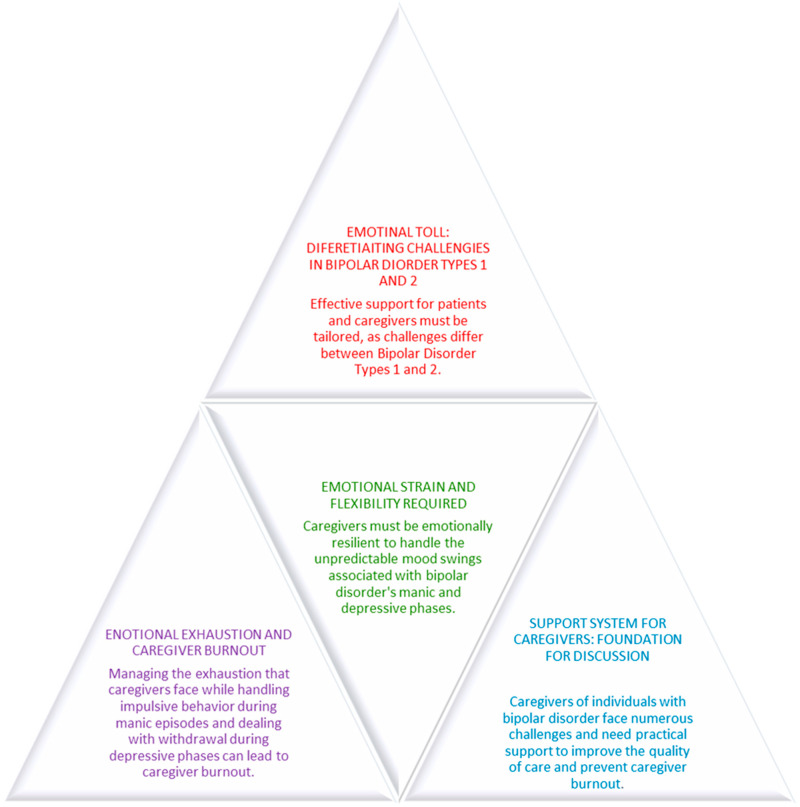
This pyramid diagram illustrates the multifaceted challenges faced by caregivers of individuals with bipolar disorder, highlighting the emotional, social, and practical aspects of caregiving. The structure is organized into three main levels to demonstrate the increasing complexity of the issues and the need for tailored support strategies.

Bipolar disorder (BD) is a complex and chronic mental health condition characterized by extreme mood fluctuations. These mood swings include manic episodes of heightened energy, impulsivity, and sometimes reckless behavior, as well as depressive episodes characterized by deep sadness, fatigue, and feelings of hopelessness [1]. These alternating states create unique challenges not only for the individuals diagnosed with BD but also for their caregivers and healthcare providers. Caregivers often experience considerable emotional and physical strain in managing both the manic and depressive symptoms [2]. This introduction will explore the complexities of BD, the unique difficulties faced by caregivers and healthcare providers, and the emotional toll that comes with managing such a dynamic and sometimes volatile condition [3]. In Figure 1, we present the context of challenges that caregivers experience while taking care of persons with bipolar disorder (Figure 1).

Base Level (Ground Level: Levels 1 and 2)

At the foundation of the pyramid, we first emphasize the need for support systems for caregivers, acknowledging the core challenge that caregivers face in managing the emotional and practical demands of caring for individuals with bipolar disorder. The emotional strain and flexibility required are highlighted here, with caregivers needing to adapt to the unpredictable nature of mood swings and be resilient in the face of constant change.

Middle Level (Level 3)

Moving up the pyramid, this level focuses on the emotional exhaustion and caregiver burnout that arises from the unpredictable and taxing nature of caregiving, from managing manic episodes to coping with depressive phases. It emphasizes the critical importance of both emotional and practical support to alleviate caregiver strain and prevent burnout.

Apex Level (Levels 4 to 6)

At the top of the pyramid, the final level addresses more specific and nuanced challenges:

Emotional demands on healthcare providers, highlighting the strain on those supporting bipolar disorder patients and the need for specialized training for healthcare professionals to recognize and address these demands.

The broader social impact of caregiver strain, showing how the emotional toll on caregivers extends to families and communities, requiring a more holistic approach to caregiving.

The differentiation of challenges between Bipolar Disorder Type 1 and Type 2, stressing the necessity of tailored caregiving strategies for each type to address the varying intensity and manifestations of the disorder.

### 1.1. The Complexity of Bipolar Disorder: A Rollercoaster of Emotions

BD is characterized by dramatic and unpredictable shifts between manic and depressive states. During manic episodes, individuals may experience euphoric feelings, increased energy, racing thoughts, and impulsive behavior, which can result in reckless decision-making. On the other hand, depressive episodes bring feelings of deep sadness, hopelessness, fatigue, and a loss of interest in daily activities. The fluctuating nature of the disorder creates challenges in care, as individuals may present very different needs depending on whether they are in a manic or depressive phase [4].

This unpredictability complicates the role of caregivers, who must constantly adapt to the changing needs of their loved ones. Caregivers must be prepared for sudden mood shifts and often feel ill-equipped to manage the intensity of emotions and behavior associated with each phase [2].

### 1.2. The Caregiver’s Struggle: Balancing Love, Support, and Stress

Caregiving for someone with BD is emotionally and physically demanding. During manic episodes, caregivers may be required to manage their loved one’s impulsivity, irritability, and disruptive behavior, which can lead to feelings of frustration and exhaustion [5]. During depressive episodes, the caregiver’s role shifts to providing emotional support, while also dealing with the overwhelming sadness and lack of motivation that their loved one may experience. These constant transitions between roles can create stress, strain relationships, and lead to caregiver burnout [5].

Caregivers also face unique emotional challenges. Feelings of guilt, anxiety, and fear of relapse can be overwhelming. Many caregivers struggle to find a balance between supporting their loved one and maintaining their own mental and emotional well-being, leading to higher rates of anxiety, depression, and chronic stress [6].

### 1.3. Healthcare Providers: The Hidden Toll of Managing Bipolar Disorder

Healthcare providers, including psychiatrists, therapists, and primary care physicians, face their own set of challenges when treating individuals with BD. While these professionals possess the expertise to treat and manage the disorder, the highly variable nature of the condition complicates treatment plans. What works during one phase of the disorder may not be effective during another, requiring constant adjustments to the treatment approach [1].

Healthcare providers are also expected to support caregivers, who often rely on them for advice and emotional guidance. The constant need to balance acute medical care with emotional support can also contribute to stress and burnout for healthcare professionals. Recognizing and addressing this hidden toll is essential to providing better care for both the individual with BD and those who are providing care.

### 1.4. The Ripple Effect: How Caregiver Strain Impacts the Larger Family and Community

The emotional and physical strain on caregivers does not affect only the immediate family member but can also have ripple effects on the broader family unit and community. Caregivers may feel isolated, with their emotional and physical resources stretched thin. This isolation can lead to relationship strain within the family, as other members may feel neglected or resentful due to the intense focus on the individual with BD [6,7].

Additionally, the strain on caregivers can increase the demand for community resources such as mental health support services, counseling, and specialized care. Communities may experience a heightened need for resources that provide support for both caregivers and individuals with BD, highlighting the importance of a collective approach to caregiving and mental health management [7].

### 1.5. Bipolar Disorder Type 1 and Caregivers: Unique Challenges

Type 1 BD is characterized by more extreme manic episodes, often involving symptoms such as psychosis, delirium, and severe impulsivity, which can place significant stress on caregivers. During manic phases, individuals with Type 1 BD may exhibit highly unpredictable behavior, making it difficult for caregivers to manage day-to-day activities or ensure their loved one’s safety. These extreme episodes require constant supervision, intervention, and often hospitalization, putting a considerable emotional and physical burden on caregivers [8].

For caregivers, the manic episodes of Type 1 BD often result in feelings of helplessness, fear, and exhaustion, as they try to control behaviors that can be dangerous or self-destructive. Conversely, the depressive episodes associated with Type 1 BD can be equally challenging, as individuals may withdraw from all social interaction and refuse to participate in daily activities. This can leave caregivers feeling isolated and emotionally drained, as they struggle to provide the care their loved one needs without adequate support or respite [9].

### 1.6. Bipolar Disorder Type 2 and Caregiver Stress: Navigating the Subtle Shifts

Type 2 BD, while less severe than Type 1 in terms of manic episodes, still presents significant challenges. Type 2 BD is characterized by hypomanic episodes, which involve elevated moods and increased energy but do not typically reach the extreme levels of mania seen in Type 1. Although hypomanic episodes are less intense, they can still lead to impulsive behavior and emotional volatility, creating stress for caregivers. Additionally, the depressive episodes in Type 2 BD can be prolonged and more difficult to detect, as individuals may try to mask their symptoms [10].

For caregivers, the challenge with Type 2 BD often lies in the subtler nature of the disorder, where symptoms may not be as easily recognizable. This can lead to caregivers feeling frustrated and confused, as they may struggle to determine whether their loved one is experiencing a mild mood shift or a more significant depressive episode. The constant fluctuations between hypomania and depression, coupled with the emotional toll of managing such shifts, can result in high levels of caregiver stress, burnout, and emotional exhaustion [7].

### 1.7. Perspectives: A Call for Greater Support and Understanding

Given the considerable strain that BD places on both individuals with the condition and their caregivers, it is essential to foster greater understanding and support for those involved in the caregiving process. Caregivers need more accessible resources, respite care, and emotional support to prevent burnout and ensure that they are able to maintain their own well-being while caring for their loved ones. Mental health professionals must also be trained to recognize the unique challenges faced by caregivers and offer strategies to help them cope with the emotional and physical demands of caregiving.

Support systems that integrate caregivers into the treatment process and provide education on managing the ups and downs of BD are vital. The healthcare system must also acknowledge the toll on caregivers and healthcare providers alike, creating environments that reduce stress and promote collaborative care models. By offering comprehensive and compassionate support for both individuals with BD and those who care for them, we can improve the overall quality of life for all parties involved.

### 1.8. Objectives

The objective of this opinion is to identify the unique emotional and behavioral challenges faced by caregivers of individuals with BD. A secondary objective is to propose targeted psychotherapeutic and educational strategies for both individual therapy sessions and tandem caregiver–patient sessions. By addressing these needs, we aim to improve the coping mechanisms of caregivers and enhance their ability to manage the emotional and physical strain associated with caregiving. These strategies will also foster better communication between caregivers and healthcare providers, ultimately contributing to a more effective management of BD.

## 2. Discussion

The discussion in this section will focus on the multifaceted challenges faced by caregivers of individuals with BD, examining the emotional strain, the complexities of caregiving, and the need for appropriate support systems. First, we will explore the unpredictable nature of BD, particularly the emotional rollercoaster of mood shifts, which requires caregivers to be flexible and emotionally resilient. This is important because understanding the volatility of the condition is crucial for caregivers to adapt to rapidly changing needs and avoid burnout. Next, we will delve into caregiver strain, highlighting the emotional exhaustion caregivers face, from managing impulsive behaviors during manic episodes to coping with the withdrawal seen in depressive phases. Recognizing these burdens is vital as it underscores the necessity for emotional and practical support to prevent burnout. Then, we will discuss the role of healthcare providers, emphasizing how the emotional demands of supporting BD patients can affect their own well-being, often going unnoticed. This is important because it calls for greater recognition and training to support both patients and caregivers effectively. Furthermore, we will explore the broader ripple effects of caregiver strain, showing how the emotional and physical toll on caregivers extends beyond the individual to impact families and communities. Understanding this broad impact is essential for fostering a more comprehensive approach to caregiving. Lastly, we will differentiate the challenges posed by Type 1 and Type 2 BD, illustrating how each requires tailored caregiving strategies. This distinction is key in providing caregivers with the specific tools they need to manage the different intensities of the disorder. Throughout, the discussion will stress the need for systemic support, education, and greater empathy from healthcare professionals and communities to ensure that caregivers can sustain their roles without compromising their own health (Table 1).

### 2.1. Unpacking the Complexity of Bipolar Disorder: A Constant Emotional Shift

BD is a mental health condition marked by extreme mood swings, from manic highs to depressive lows. These shifts create a complex environment not only for the person diagnosed but also for their caregivers. During manic episodes, the individual may exhibit elevated energy levels, impulsive behavior, and a lack of judgment, all of which require heightened vigilance from caregivers. Conversely, the depressive episodes are characterized by overwhelming sadness, hopelessness, and an inability to engage with daily life, creating a stark contrast in caregiving approaches. The unpredictable nature of BD’s mood shifts makes managing care particularly challenging, as caregivers must remain flexible and responsive to each phase’s distinct needs [1].

Caregivers, caught between these extremes, often face emotional strain as they adjust to rapidly changing behaviors. For instance, a caregiver may spend the day managing an emotionally energetic, impulsive loved one during a manic episode, only to shift to a quieter, emotionally withdrawn environment during a depressive episode. This constant switching demands not only emotional flexibility but also resilience. Unfortunately, this unpredictability can lead to emotional burnout as caregivers struggle to maintain a sense of stability. They may feel overwhelmed by the lack of predictability and find it difficult to focus on their own emotional health. For many, the need to constantly adjust their expectations and caregiving strategies contributes to a sense of uncertainty and emotional exhaustion. This cyclical nature of BD demands that caregivers develop coping mechanisms, access resources, and establish support systems to better manage the constant emotional shifts that are a hallmark of the condition [11].

Thus, the complexity of BD requires caregivers to adopt a multi-faceted approach, one that encompasses both emotional and practical aspects of care. Understanding the disorder’s unique challenges is essential for developing effective strategies that ensure that caregivers can respond to their loved ones’ changing emotional states without compromising their own mental health.

### 2.2. Caregiver Strain: Navigating the Emotional Burden of Bipolar Disorder

Caregivers of individuals with BD often endure immense emotional strain as they cope with the continuous fluctuations of mood between manic highs and depressive lows [3]. One of the most significant challenges faced by caregivers is the emotional exhaustion that comes from managing these shifts. During manic episodes, caregivers are often tasked with controlling impulsive and sometimes dangerous behavior, which can result in feelings of frustration, anxiety, and helplessness. For example, when an individual is overly energetic or engages in risky behavior, caregivers may feel overwhelmed by the need to intervene quickly to prevent harm. In contrast, during depressive episodes, caregivers may struggle to motivate their loved ones, witnessing their emotional withdrawal and feeling powerless in the face of their loved one’s suffering [12].

This dual emotional burden—dealing with both the heightened emotionality of manic phases and the low energy of depressive episodes—can take a toll on caregivers’ mental health. Many caregivers report feeling isolated and unable to share their challenges with others, contributing to a sense of loneliness and alienation. Guilt can also play a significant role in the emotional strain, as caregivers may blame themselves for not being able to “fix” their loved one’s condition or help them recover. Over time, this sense of helplessness can lead to caregiver burnout, which manifests as chronic stress, fatigue, and emotional exhaustion. The caregiving role often leaves little time for self-care, and many caregivers neglect their own health, both physical and emotional, which compounds the stress they already experience [3,12].

It is crucial for caregivers to receive emotional and practical support to help mitigate these challenges. Support groups, mental health counseling, and respite care are important resources that can provide caregivers with the tools and emotional relief they need to continue in their caregiving role. Without such support, the strain can become overwhelming, leading to both caregiver and patient experiencing negative outcomes [2].

### 2.3. Healthcare Providers’ Role: The Invisible Burden of Bipolar Disorder Management

Healthcare providers who treat individuals with BD play a critical role in managing both the medical and emotional aspects of the condition. However, the strain on these professionals is often not fully recognized. In addition to addressing the pharmacological needs of individuals with BD, healthcare providers must navigate the emotional challenges associated with managing a condition that involves frequent mood swings. During manic episodes, individuals may resist treatment or refuse to follow medical advice, which can cause additional frustration for both the patient and the healthcare provider. This resistance can increase the emotional burden on providers, as they must remain patient and persistent while also offering emotional support [13].

Similarly, when individuals are in a depressive episode, healthcare providers must be aware of the emotional toll it takes on both the individual and their caregivers. Providers often have to walk a fine line between being medically directive and offering empathetic emotional support. This balancing act can be taxing, especially when caregivers are seeking guidance on how to handle the emotional impact of their loved one’s mood swings. As caregivers often rely heavily on healthcare professionals for advice and reassurance, the emotional labor associated with their role can contribute to provider burnout [13,14].

Moreover, healthcare providers may not always have access to the resources needed to provide comprehensive support to caregivers. Training in the emotional aspects of caregiving, as well as in recognizing signs of caregiver stress, can equip providers with the necessary skills to offer both medical and emotional support [2]. By addressing both the physical and emotional needs of caregivers and patients, healthcare providers can contribute significantly to improving the well-being of everyone involved in the caregiving process.

### 2.4. Ripple Effects: The Broader Impact of Caregiver Strain on Families and Communities

The emotional and physical toll of caregiving for someone with BD extends beyond the immediate caregiver to affect the entire family and broader community. Caregivers are often forced to dedicate so much of their time and energy to their loved one that their own personal relationships suffer. In families where one member is primarily responsible for caregiving, other members may feel neglected or frustrated, leading to strained relationships and communication breakdowns. This dynamic can further isolate the caregiver, as they may feel misunderstood or unsupported by other family members who may not fully grasp the demands of caregiving [15].

Furthermore, the emotional exhaustion experienced by caregivers often leads to a decrease in their social interactions, leaving them disconnected from their social networks. This social isolation can exacerbate the caregiver’s mental health struggles, leading to feelings of loneliness, depression, and anxiety. This disconnect is compounded by the fact that many caregivers are reluctant to ask for help or may feel that they are not entitled to take time for themselves, fearing that it will negatively impact their loved one’s well-being [16].

The impact of caregiver strain extends beyond the family unit, affecting the broader community as well. Caregivers may need additional community-based support services, including mental health resources, respite care, and educational programs. If the community fails to recognize the strain placed on caregivers, the emotional and physical toll will likely continue to increase. A more robust support system—one that encourages caregiver engagement, offers practical help, and fosters awareness—can help mitigate the strain experienced by caregivers, ultimately benefiting not just the caregiver but also the entire family and community [17].

### 2.5. Unique Caregiving Challenges in Bipolar Disorder Type 1: Extreme Emotional Shifts

Type 1 BD, known for its more severe manic episodes, presents unique challenges for caregivers that differ from those of Type 2. The manic episodes in Type 1 can escalate quickly, with individuals engaging in high-risk behaviors that may endanger themselves or others. For example, during manic episodes, individuals may exhibit poor judgment, engage in reckless spending, or make impulsive decisions that have long-term consequences. Caregivers must be on high alert to prevent dangerous situations, which can cause immense emotional strain and anxiety. The unpredictable nature of these episodes can lead to a sense of chaos, making it difficult for caregivers to maintain any sense of control or stability in their caregiving role [10,18].

Furthermore, the depressive episodes that follow manic phases can leave the individual with BD completely incapacitated, requiring caregivers to provide intensive support. The contrast between the high-energy mania and the deep despair of depression creates an emotional rollercoaster for caregivers, who must continually adjust their caregiving strategies to accommodate the changing needs of their loved one. These emotional fluctuations often result in caregiver burnout, as the caregiver may feel that they are constantly navigating between extremes without respite [2,7,11].

Healthcare providers and caregivers must be particularly vigilant when managing Type 1 BD. The severity of manic episodes, combined with the incapacitation caused by depressive episodes, makes it essential that caregivers are adequately supported and equipped with strategies for managing both phases of the disorder. Addressing the emotional and practical needs of caregivers is crucial in ensuring their ability to sustain long-term care for individuals with Type 1 BD [18].

### 2.6. Subtle Shifts in Bipolar Disorder Type 2: Navigating the Nuances of Caregiving

Type 2 BD, though often considered less severe than Type 1, presents unique challenges for caregivers that can be just as emotionally taxing. In Type 2, individuals experience hypomanic episodes, which are less extreme than the manic episodes seen in Type 1, but they still result in impulsive behavior and emotional instability. The subtle nature of hypomania makes it harder for caregivers to identify the onset of a mood shift, often leading to confusion and frustration. Unlike the dramatic mood swings of Type 1, the shifts in Type 2 BD can be more insidious, requiring caregivers to be highly attuned to their loved one’s behavior in order to recognize when a change is occurring [19].

The prolonged depressive episodes in Type 2 BD also present a unique set of challenges. While the depression in Type 1 can be acute and more easily identified, the depression in Type 2 is often more subtle and enduring. Caregivers may struggle to detect the onset of depressive symptoms, which can lead to prolonged periods of emotional withdrawal that are difficult to manage. This extended period of depression can leave caregivers feeling helpless and emotionally drained, as they are unable to provide the level of support that their loved one needs [19,20].

Although Type 2 BD is often characterized by less extreme mood fluctuations, the persistence of these symptoms requires careful monitoring and continuous support. For caregivers, this can lead to a sense of emotional fatigue, as they navigate the complexities of recognizing and managing these more subtle shifts. To address these challenges, caregivers must be educated about the signs of hypomania and depression and be provided with tools to manage the emotional demands of caregiving for individuals with Type 2 BD.

### 2.7. A Call for Greater Support and Understanding: Perspectives on Caregiving for Bipolar Disorder

The unique challenges faced by caregivers of individuals with BD cannot be overstated. Caregiving for someone with this condition is an emotionally demanding role that requires significant support, both for the caregiver’s well-being and for the effective management of the disorder itself. It is critical for healthcare providers to recognize the immense emotional labor involved in caregiving and to offer resources that help mitigate the strain on caregivers. This includes providing mental health support, respite care, and opportunities for caregivers to connect with others in similar situations through support groups [21].

The broader community also plays a crucial role in alleviating caregiver stress. Increased awareness and education about BD can help foster empathy and reduce the stigma often associated with mental health conditions. By building a network of support comprising healthcare professionals, family members, and community organizations, caregivers can be better equipped to manage the complexities of their role without sacrificing their own well-being. Thus, caregiving for individuals with BD requires a comprehensive support system that recognizes both the medical and emotional aspects of care. Only through greater understanding, education, and systemic support can caregivers truly thrive in their role while ensuring the best outcomes for both themselves and their loved one’s various issues and challenges that caregivers of individuals with BD face [2,22]. In Table 2, we present a holistic care approach for bipolar disorder addressing caregiver needs and improving care outcomes.

### 2.8. What Now? Addressing the Gaps in Caregiving for Bipolar Disorder

After exploring the unique challenges faced by caregivers of individuals with BD, it becomes clear that addressing these challenges requires concerted effort and practical solutions. This “What Now?” section outlines potential avenues for improving the caregiving experience, advocating for systemic changes, and highlighting the importance of support mechanisms for both caregivers and healthcare providers.

#### 2.8.1. Call for Further Research

While significant strides have been made in understanding BD, there remains a critical need for further research into the specific impacts of caregiving on both the mental and physical health of caregivers. Most existing studies tend to focus on the clinical management of BD, leaving a gap in the literature regarding the strain on caregivers. Further research could explore the long-term consequences of caregiving, including chronic stress, anxiety, depression, and even physical health issues such as cardiovascular problems linked to high caregiver stress. Additionally, longitudinal studies would be valuable in understanding how caregiving for individuals with BD evolves over time, especially in relation to the different phases of the disorder, and how the emotional toll changes as the caregiver and patient age. By focusing on the experiences and needs of caregivers, research can inform the development of targeted interventions and support programs. There is also an opportunity to explore whether certain caregiving strategies are more effective in preventing burnout or managing stress. Ultimately, more research into this underexplored area can pave the way for evidence-based solutions that improve the well-being of caregivers and those they care for [15,23].

#### 2.8.2. Advocacy for Caregiver Support

Advocacy for caregiver support is crucial in addressing the overwhelming emotional and physical strain that caregivers experience. Policymakers and mental health organizations must recognize the significant burden of caregiving for individuals with BD and work to create more formalized support structures. This might include expanding access to respite care, which provides caregivers with temporary relief from their responsibilities. Respite care allows caregivers to take a break, attend to their own health, or engage in activities that recharge them emotionally. Another critical area for advocacy is financial support for caregivers, as many find themselves in situations where they cannot work full-time due to their caregiving responsibilities. Financial compensation or tax relief for caregivers could alleviate some of the stress and financial strain they face. Mental health support should also be integrated into the caregiving experience, with caregivers being offered access to counseling or therapy specifically designed to help them manage stress, anxiety, and feelings of guilt. Advocacy efforts should also focus on reducing the stigma surrounding caregiver exhaustion, as many caregivers suffer in silence due to feelings of shame or a belief that they must remain strong for their loved one. By highlighting the importance of caregiver well-being in public policy discussions and within the healthcare system, we can ensure that caregivers receive the support they need to thrive in their roles [11,16,23,24].

#### 2.8.3. Implementation of Training Programs for Caregivers

One of the most effective ways to support caregivers is through the implementation of specialized training programs. Caregivers need not only emotional support but also practical skills to manage the daily challenges associated with BD. A comprehensive training program could cover a variety of topics, including understanding the symptoms of manic and depressive episodes, how to de-escalate potentially harmful situations during manic phases, and effective communication strategies to support someone with BD during depressive episodes. Such programs could be delivered through online courses, local workshops, or community health centers. Training should also focus on teaching caregivers how to recognize the signs of caregiver burnout and provide them with tools to prevent or manage it. This might include stress management techniques, time management skills, and coping mechanisms such as mindfulness and relaxation exercises. Additionally, caregivers should be educated about the resources available to them, such as support groups, respite care, and online forums where they can share experiences with others in similar situations. Ultimately, providing caregivers with the knowledge and skills to navigate the complexities of BD will empower them to provide better care while safeguarding their own well-being. This proactive approach to caregiver education will ensure that caregivers feel more confident and less isolated in their role [5,25,26].

#### 2.8.4. Improved Communication Between Caregivers and Healthcare Providers

Effective communication between caregivers and healthcare providers is essential for managing BD. Caregivers are often on the frontlines, dealing with the day-to-day realities of the disorder, yet they may not always have access to the medical advice and support they need. Healthcare providers should foster an open, two-way communication channel with caregivers, where caregivers feel comfortable sharing their observations, concerns, and questions. Regular check-ins, either in person or through telemedicine, could help keep caregivers informed and allow healthcare providers to adjust treatment plans as needed. It is also essential that healthcare professionals understand the emotional labor involved in caregiving and offer empathy, recognizing that caregivers are often dealing with significant stress and emotional challenges. This understanding can help prevent caregiver burnout and improve the overall caregiving experience. Furthermore, caregivers should be empowered to actively participate in treatment decisions, as they often have valuable insights into the patient’s behavior and emotional state that could influence the effectiveness of medication and therapy. Promoting a collaborative approach, where caregivers and healthcare providers work together as a team, ensures more holistic care for individuals with BD and better outcomes for both patients and caregivers [23,26,27].

#### 2.8.5. Community-Based Solutions

A critical element in supporting caregivers of individuals with BD is the involvement of the broader community. Many caregivers experience social isolation as they prioritize the needs of their loved ones over their own social connections. Community-based support solutions can help mitigate this isolation and provide caregivers with the resources they need to sustain their roles. Community support groups for caregivers can provide a space where caregivers can share their experiences, receive emotional support, and learn coping strategies from others in similar situations. In addition, community education programs can raise awareness about BD and reduce the stigma surrounding mental health. This could help generate empathy from the wider public and encourage more community involvement in supporting caregivers. Local mental health organizations and social services can play a key role in connecting caregivers with services such as respite care, counseling, and legal or financial assistance. Furthermore, public spaces like churches, schools, or community centers could host events that bring together caregivers for social and educational purposes. By creating a more supportive community environment, caregivers can feel less isolated and more empowered to manage the demands of their caregiving role [23,28].

#### 2.8.6. Holistic Care Approaches

The final point addresses the need for a holistic approach to caregiving for individuals with BD, one that considers both the medical and emotional needs of caregivers. While medical treatments for BD are essential, they are not enough on their own to address the full spectrum of challenges caregivers face. A holistic care approach would incorporate mental health support for caregivers, stress management resources, and ongoing education to help them navigate the emotional rollercoaster of caregiving. This approach might include therapy sessions, mindfulness practices, and physical health resources, such as exercise programs or relaxation techniques, to help caregivers manage the physical and mental toll of their role. It could also involve providing caregivers with access to peer support networks, where they can connect with others who understand their unique challenges. By addressing both the practical and emotional needs of caregivers, a holistic approach ensures that caregivers are not only able to provide effective care to their loved ones but also maintain their own well-being. This comprehensive model would require collaboration between healthcare providers, community organizations, and policymakers to ensure that caregivers are fully supported in their critical role [5,25,26,29].

## 3. Conclusions

In conclusion, it is evident that caregivers of individuals with BD face distinct emotional and behavioral challenges due to the unpredictable nature of the disorder’s mood swings. The constant shift between manic and depressive episodes creates an environment of emotional instability, placing a significant strain on caregivers’ mental and physical well-being. These challenges often lead to burnout, stress, and a sense of isolation, making it crucial to address the unique needs of caregivers through targeted psychotherapeutic and educational strategies.

We conclude that providing caregivers with individualized therapy, such as Cognitive Behavioral Therapy and mindfulness techniques, can help them manage stress and improve coping mechanisms. Furthermore, psychoeducation is vital in equipping caregivers with the necessary knowledge about BD and its impact on both the patient and the caregiver. Tandem caregiver–patient therapy sessions also play a critical role in fostering communication, empathy, and mutual understanding, allowing caregivers to better navigate the emotional and behavioral complexities of the disorder. These strategies not only help caregivers improve their emotional resilience but also enhance their ability to manage caregiving responsibilities without compromising their own well-being.

Ultimately, by implementing these approaches, caregivers can build stronger support systems, reduce emotional exhaustion, and improve their overall caregiving experience. Additionally, promoting better communication between caregivers and healthcare providers can lead to a more effective management of BD, benefiting both the caregiver and the individual with the condition. Thus, addressing the emotional and behavioral challenges faced by caregivers is essential for improving the quality of care and well-being for all parties involved.

## Figures and Tables

**Table 1:** The difficulties faced by caregivers of individuals with bipolar disorder and proposed corresponding strategies that can be used in therapy sessions for both the caregiver alone and in tandem with the patient.

Emotional and Behavioral Challenges	Targeted Psychotherapeutic and Educational Strategies	Individual Therapy (Caregiver)	Tandem Caregiver–Patient Therapy
Emotional Strain	Psychotherapy focusing on stress management, anxiety reduction, and emotional resilience.	- Cognitive Behavioral Therapy (CBT) to address anxiety, guilt, and grief. - Mindfulness techniques for emotional regulation.	- Joint mindfulness and relaxation exercises to foster mutual emotional understanding.
Burnout and Fatigue	Psychoeducation on caregiving burden and establishing self-care routines.	- Psychoeducation on recognizing signs of burnout. - Self-compassion.	- Training on sharing caregiving responsibilities and reducing burnout through joint goal-setting.
Isolation and Loneliness	Developing social support networks and encouraging connection with peer caregivers.	- Support group participation or one-on-one counseling with other caregivers.	- Facilitating joint sessions where both caregiver and patient engage in social activities.
Frustration and Helplessness	Addressing the feelings of powerlessness and exploring ways to promote empowerment.	- Empowerment-based therapy to shift focus from helplessness to problem-solving.	- Engaging both caregiver and patient in collaborative problem-solving exercises.
Difficulty Managing Mood Swings	Teaching communication techniques and coping strategies to handle mood episodes.	- Psychoeducation on bipolar disorder and coping strategies. - Role-playing to handle extreme mood episodes.	- Simulating common caregiving scenarios to practice de-escalation strategies.
Fear of Relapse or Crisis	Psychoeducation on managing crises and preparing for potential relapses.	- Crisis planning and proactive coping strategies.	- Joint crisis intervention planning with patient and caregiver involvement.
Guilt and Self-Blame	Addressing cognitive distortions and fostering self-compassion.	- Cognitive Behavioral Therapy (CBT) to challenge guilt-related thoughts.	- Building a narrative of shared responsibility and mutual understanding in the caregiving role.

**Table 2:** Holistic care approach for bipolar disorder: addressing caregiver needs and improving care outcomes.

Key Element	Caregiver Support Strategies	Expected Outcome
Emotional Well-Being of Caregivers	- Training on stress management and emotional resilience.	- Reduced caregiver burnout and emotional exhaustion.
	- Psychoeducation on managing caregiver stress.	- Improved emotional resilience to manage caregiving challenges.
Mood Episode Management	- Training caregivers on how to manage mood episodes (both manic and depressive).	- Caregivers are better equipped to handle unpredictable mood swings.
	- Developing crisis management plans.	- Improved patient care during episodes.
Peer Support Networks	- Encouraging caregiver participation in peer support groups.	- Increased emotional support for caregivers.
	- Facilitating communication with other caregivers.	- Strengthened sense of community and shared understanding.
Access to Mental Health Services	- Offering caregivers counseling and access to mental health services.	- Improved caregiver mental health.
	- Providing referral services to mental health professionals.	- Greater ability to cope with caregiving challenges.
Improved Quality of Life (QOL)	- Providing a balanced approach to care, including emotional support and practical resources.	- Enhanced well-being for both caregivers and patients.
	- Encouraging self-care practices for caregivers.	- Improved caregiving experience and overall quality of life.
Collaborative Care Approach	- Including both the patient and caregiver in the treatment plan.	- More effective care coordination.
	- Fostering open communication between caregivers, patients, and healthcare providers.	- Better outcomes for both patients and caregivers due to shared involvement in care.

## Data Availability

The original contributions presented in the study are included in the article, further inquiries can be directed to the corresponding author.

## Abbreviations

The following abbreviations are used in this manuscript:

BD	Bipolar disorder
